# Snake‐Eye Screwing: A Novel Free‐Hand Technique of Pedicle Screw Placement in Cervicothoracic Spine and Preliminary Clinical Results

**DOI:** 10.1111/os.12809

**Published:** 2020-12-06

**Authors:** Jian Zhu, Kai‐qiang Sun, Lan‐tao Lu, Jing‐chuan Sun, Yong‐fei Guo, Yuan Wang, Qing‐jie Kong, Xi‐ming Xu, Jian‐gang Shi

**Affiliations:** ^1^ Department of Orthopaedic Surgery, Spine Center, Changzheng Hospital Second Military Medical University Shanghai China; ^2^ Ninth Team, Basic Medical College Naval Medical University Shanghai China

**Keywords:** Cervicothoracic spine, Complications, Free‐hand, Pedicle screw

## Abstract

**Objective:**

To propose a novel technique of free‐hand pedicle screw placement in cervicothoracic spine (snake‐eye method) and evaluate the preliminary effects and safety in clinical practice.

**Methods:**

This is a retrospective study and we defined the period of this study as from December 2017 to April 2019 in our institution. Forty patients were included in this study who underwent cervicothoracic internal fixation in our hospital, and all patients undergoing implantation of 200 pedicle screws were divided into two groups. Twenty‐two patients (108 screws) had screw placement using traditional method, while 18 patients (92 screws) had screw placement using snake‐eye method. To reduce the possible selection bias, the patients we recruited in this study was originally performed on by one radiological doctor who was blind to the objective of this study. Patient demographics, including patient age, sex, obesity, smoking, and hypertension, were evaluated to figure out baseline differences between groups. Medical information was recorded including time, accuracy, and immediate (within 30 days after surgery) postoperative complications of pedicle screw placement (including pulmonary embolism or other thromboembolic events, surgical site infection, neurovascular injury, and mortality).

**Results:**

There were 24 males and 16 females, with an average age of 52.2 years (range, 24–77). Finally, a total of 200 screws were successfully inserted in these patients, including fifteen patients with four pedicle screws, four patients with six screws, three patients with eight screws in traditional method group, and 12 patients with four pedicle screws, two patients with six screws, four patients with eight screws in snake‐eye method group. Patient demographic and comparison of two surgery methods are shown in Tables 1 and 2. The data baselines of the two groups were comparable because no impact of the two groups on population characteristics was demonstrated in the presented experiment. Also, we noticed that time and accuracy of the two groups were different with statistical significance at the level of *P* = 0.05. We observed that immediate (within 30 days after surgery) postoperative complications, including pulmonary embolism (PE), surgical site infection (SSI), neurovascular injury (NI), and mortality, in the two groups did not differ.

**Conclusion:**

This study highlights a safe and effective technique for pedicle screw placement in cervicothoracic spine named snake‐eye method, and this technique may be particularly useful in emergency conditions with limited resources.

## Graphical abstract

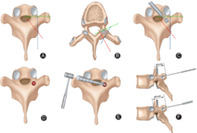

## Introduction

Pedicle screw placement is the most fundamental procedure for spine surgeons in posterior spinal reconstruction surgery, and has been shown by biomechanical studies to provide stronger stability than other posterior cervical fixation methods^1^. Numerous studies have demonstrated that the technique of free‐hand pedicle screw fixation is an effective and reliable method^2^, ^3^. Since its inception, lots of research efforts have been made to improve the speed and accuracy of pedicle screw fixation. According to the measurements of anatomical parameters, a variety of pedicle screw placement strategies have been devised and assessed. Computer‐assisted surgery has influenced traditional screw placement methods, especially in high‐risk patients with anatomical abnormalities. Although computer‐assisted surgeries can be an option for pedicle screws since they may be biomechanically better than free‐hand techniques, these surgeries have a higher risk of vascular and neural injury. Many navigation systems, including pre‐ and intra‐operative 3‐D computed tomography (CT) image navigation (CTNav), fluoroscopy‐assisted (FA) techniques, and robot‐assisted (RA) techniques, have been introduced for pedicle screw placement. However, there are still high risks in placing screws in cervicothoracic segments where perforations frequently happened. We believe that the basic surgical anatomy of the cervical and thoracic vertebrae is the key factor causing the perforations: (i) Cervicothoracic segments (C_6_–T_6_) are in deep position with complex adjacent structures, including large vessels (such as the aortic arch), trachea, esophagus, thoracic duct, recurrent laryngeal nerve, sympathetic nerve, etc^4^, ^5^, ^6^. (ii) The cervicothoracic segments transit from cervical vertebrae with a high level of mobility to relatively fixed thoracic vertebrae, where the lordosis becomes kyphosis gradually^7^. Special morphological features of cervicothoracic segments make it vulnerable to injury and cause difficulties in intraoperative exposure of visual field and localization^8^. (iii) Moreover, diameters of cervicothoracic pedicles are quite small when compared with lumbar pedicles, making it quite difficult to put the screw without peaking out. T_4_ has the smallest pedicle diameter from anatomical studies. Therefore, difficulties and risks of pedicle screwing in the cervicothoracic segments is much greater than that in other segments of the spine.

Free‐hand technique is still the most commonly used method to place screws, especially in emergency and limited situations. Currently, Albumin method^9^, Jeanneret method^10^, fenestration^11^ and funnel method^12^ are commonly used methods of free‐hand pedicle screw placement in cervicothoracic spine, which provide valuable clinical references. Pedicle screw placement accuracy of these different methods has been exhaustively reported, because accuracy is indispensable for achieving surgical success. These traditional methods include two key steps: finding the entry point and the angle of the screw trajectory. Taking the Jeanneret method as an example, the entry point is 3 mm from the caudal‐side of the intersection of the transverse process and the superior articular process. And screw trajectories are perpendicular to the sagittal plane, and 30° abducent for the midline. The rate of pedicle screw malposition has been reported from 5% to 41% in the cervical spine and from 3% to 55% in the thoracic spine^13^, ^14^, ^15^, ^16^, ^17^. Also, the varied trajectories make it quite a challenge for young spine surgeons, prolonging the learning curve. Thus, free‐hand screwing is still a great challenge in cervicothoracic spine.

It is important to examine how to place screws into the pedicles of cervicothoracic vertebrae safely and simply. Therefore, the aims of this study are: (i) to propose a novel technique of free‐hand pedicle screw placement (snake‐eye method) in cervicothoracic spine (the procedure diagram and customized surgery instruments are described in detail); and (ii) to evaluate the effects and safety of snake‐eye method in clinical practice.

## Materials and Methods

### *Patient Information*

From December 2017 to April 2019, 40 patients were included in this study who underwent cervicothoracic internal fixation in our hospital, and all patients undergoing implantation of 200 pedicle screws were divided into two groups. Twenty‐two patients (108 screws) had screw placement using traditional method, while 18 patients (92 screws) had screw placement using snake‐eye method. There were 24 males and 16 females, with an average age of 52.2 years (range, 24–77). Twenty‐six patients were treated for cervicothoracic trauma, and the other 14 patients included nine cases of cervical spondylotic myelopathy (CSM), four cases of disc herniation, and one case of cervical spondylotic radiculopathy (CSR), respectively.

Finally, a total of 200 screws were successfully inserted in these patients, including fifteen patients with four pedicle screws, four patients with six screws, three patients with eight screws in traditional method group, and 12 patients with four pedicle screws, two patients with six screws, and four patients with eight screws in snake‐eye method group.

### *Surgical Technique*

**Fig. 1:**
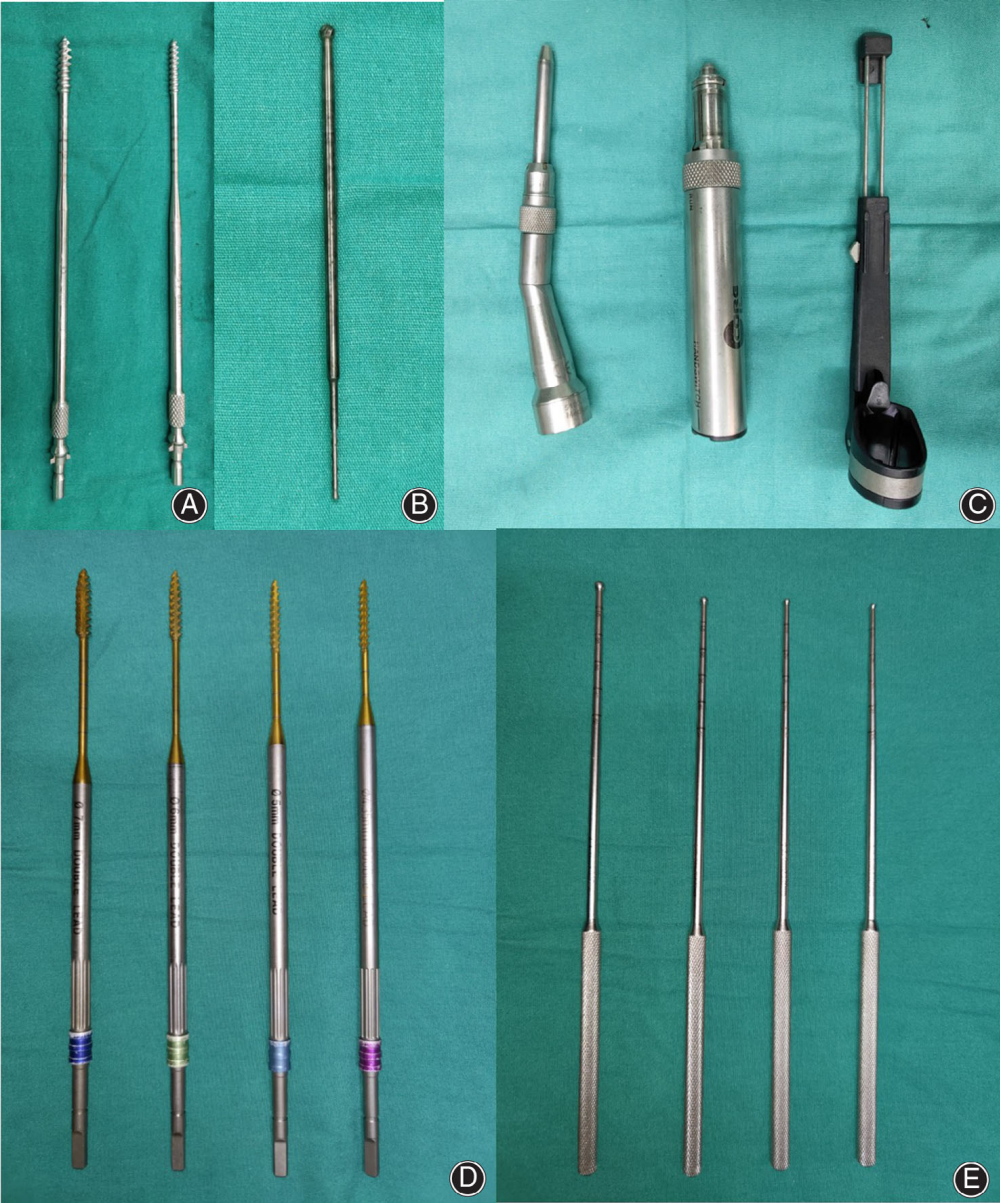
Key instruments in surgery. (A) Common screw tap. (B) 2 mm electric drill grinding head. (C) Cortical bone grinding head, electric drill power transmission device and control switch. (D) Customized screw tap. (E) Customized blunt‐headed opener.

**Fig. 2:**
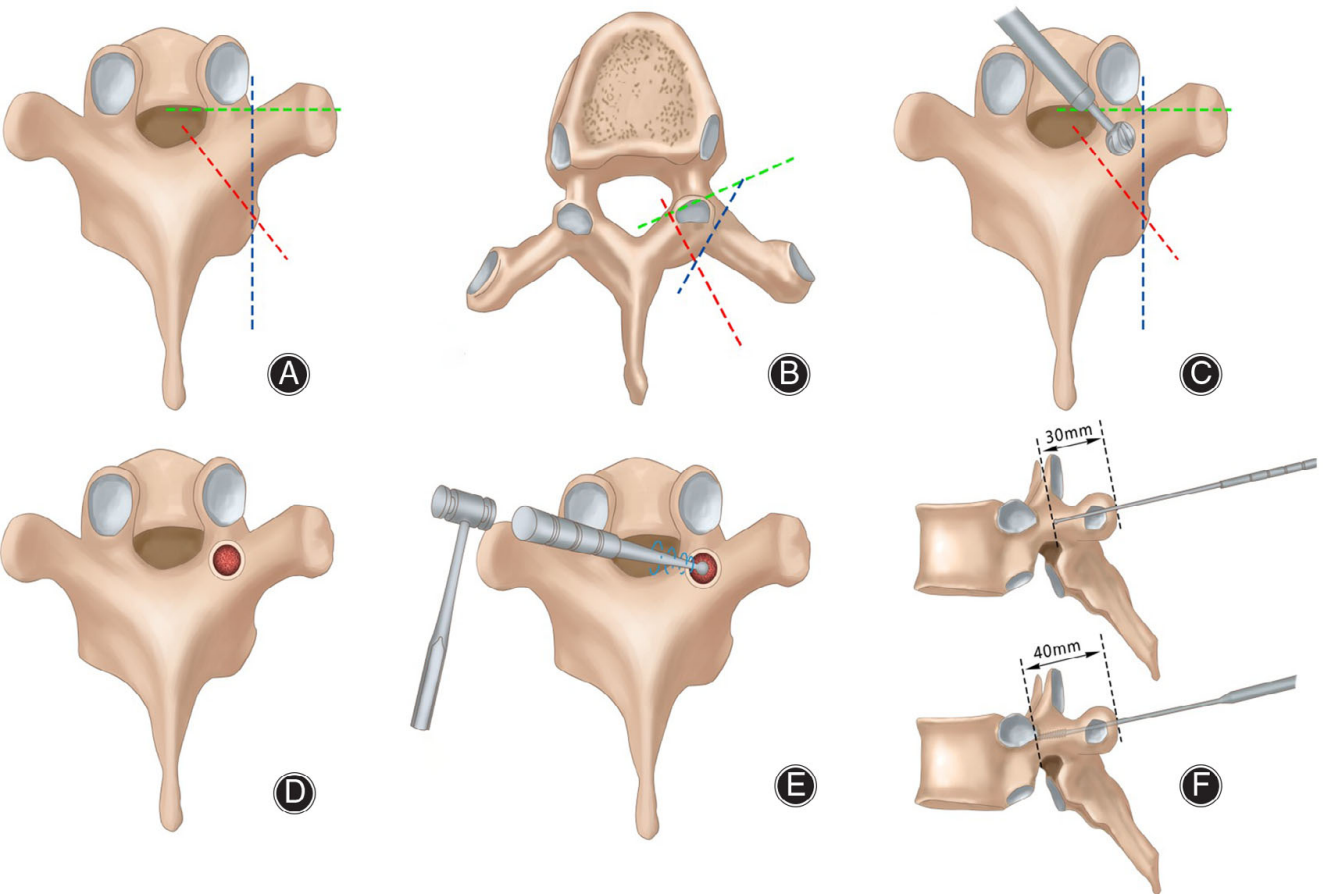
(A, B) The entry point is positioned in the middle of the triangle formed by the lower edge of the superior articular facet, the medial edge of the transverse process and the pars interarticularis. (C) The surrounding cortical bone was removed with a rongeur and a high‐speed burr, and then the cancellous bone was exposed. (D) Cortical bone lacks blood supply and appears white while cancellous bone is rich in blood supply and appears bright red, the white cortical bone around and the bright red cancellous bone in the middle are shaped like “snake eyes.” (E) The blunt‐headed opener designed and manufactured by ourselves was used to enter the vertebral pedicle with a hammer in the position of the snake eye. (F) After entering the depth of about 30 mm, the trajectory is detected with a probe in case of perforation, then the screw tap is then applied to insert approximately 40 mm along the pedicle cancellous bone.

The main surgical instruments are pedicle screws, customized screw tap, cortical bone grinding head, electric drill power transmission device, control switch, and 2 mm electric drill mushroom grinding head (Fig. 1). Bilateral lamina and facet joint were exposed using a standard posterior midline approach under general anesthesia and prone position; three frames of references were determined to provide an optimal method for establishing pedicle screw entry point, that is, the root of the transverse process, the pars interarticularis, and superior articular facet. By finding these anatomical markers, we had a frame of reference that can be used to determine the appropriate entry point for pedicle screw placement. The entry point is positioned in the middle of the triangle formed by the lower edge of the superior articular facet, the medial edge of the transverse process, and the pars interarticularis (Fig. 2A,B).

The surrounding cortical bone was removed with a rongeur and a high‐speed burr, and then the cancellous bone was exposed (Fig. 2C). Cortical bone lacks blood supply and appears white while cancellous bone is rich in blood supply and appears bright red, the white cortical bone around and the bright red cancellous bone in the middle are shaped like “snake eyes” (Fig. 2D).

**Fig. 3:**
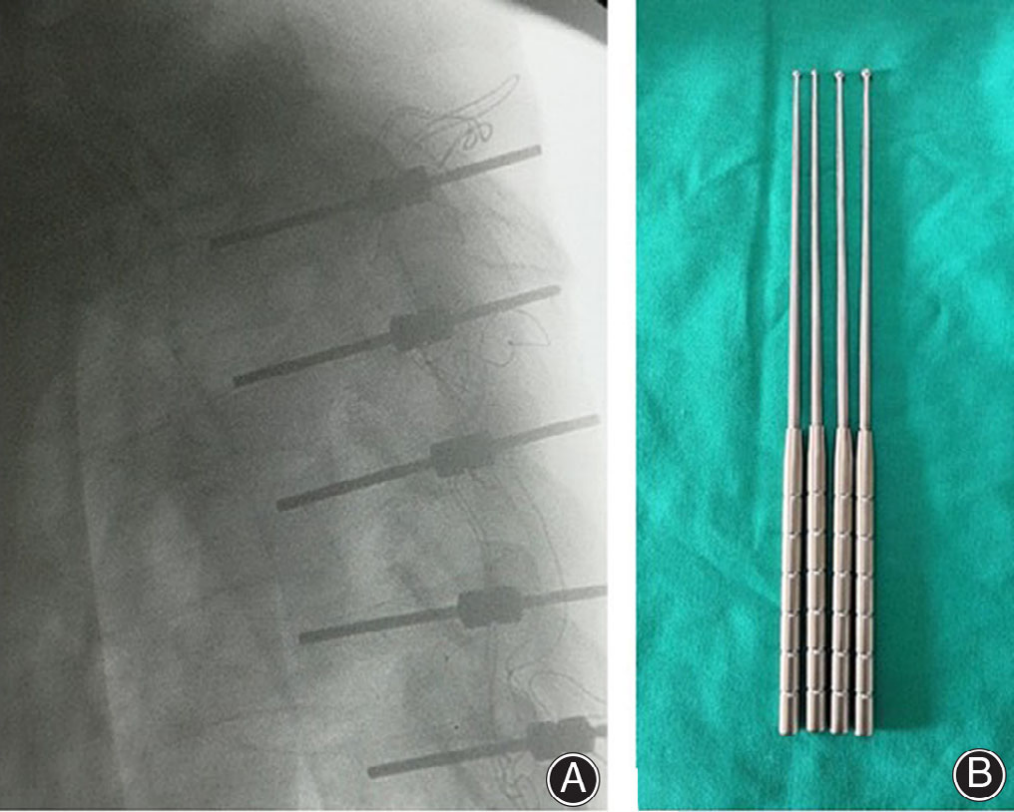
Designed blunt opener and intra‐operative X‐ray examination of markers. (A) an intra‐operative X‐ray film of markers in T_1_‐T_5_ pedicles after constructing the trajectory by snake‐eye method, indicating all markers in excellent position; (B) the designed opener with a ball tip of varied diameter.

**Fig. 4:**
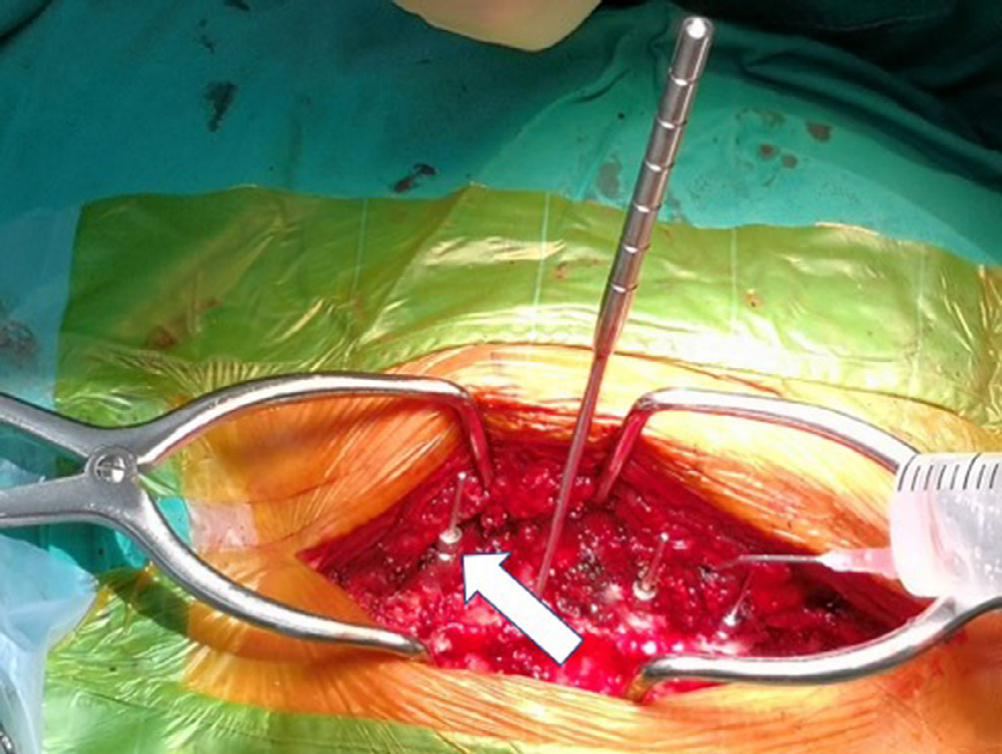
Hammering blunt opener into the pedicle. The white narrow indicates blunt opener, square indicates marker which is inserted into the trajectory constructed by the opener.

The blunt‐headed opener designed and manufactured by ourselves was used to enter the vertebral pedicle with a hammer in the position of the snake eye (Figs 2E, 3). After entering the depth of about 30 mm, the trajectory is detected with a probe in case of perforation (Fig. 2F). We moved forward along the cancellous bone during path‐making without any drastic progression which may prevent breaching. The screw tap is then inserted approximately 40 mm along the pedicle cancellous bone (Fig. 2F). A blunt probe was applied again to explore the trajectory to ensure intactness of the cortical bone (Fig. 4). After constructing the screw trajectories, a screw with a diameter of 3.5 mm or 4.0 mm and an appropriate length was then implanted with the axis of the screw consistent with the axis of the pedicle.

### *Collection of Evaluation Indicators*

#### *Patient Demographics*

Patient demographics including patient age, sex, obesity, smoking, and hypertension were evaluated to figure out baseline differences between groups. Medical information was recorded including time, accuracy, and complications of pedicle screw placement. Time of screwing was recorded for each surgery. Postoperative computed tomography (CT) and X‐ray was performed to evaluate the accuracy.

### *Rampersaud Criterion*

**Fig. 5:**
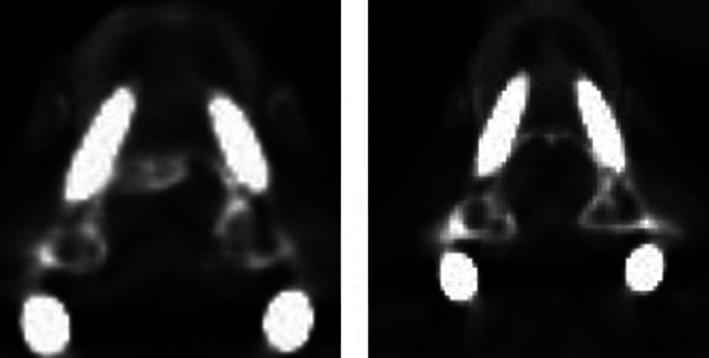
Accurate insert positioning: no bleach and less than 2 mm breach.

**Fig. 6:**
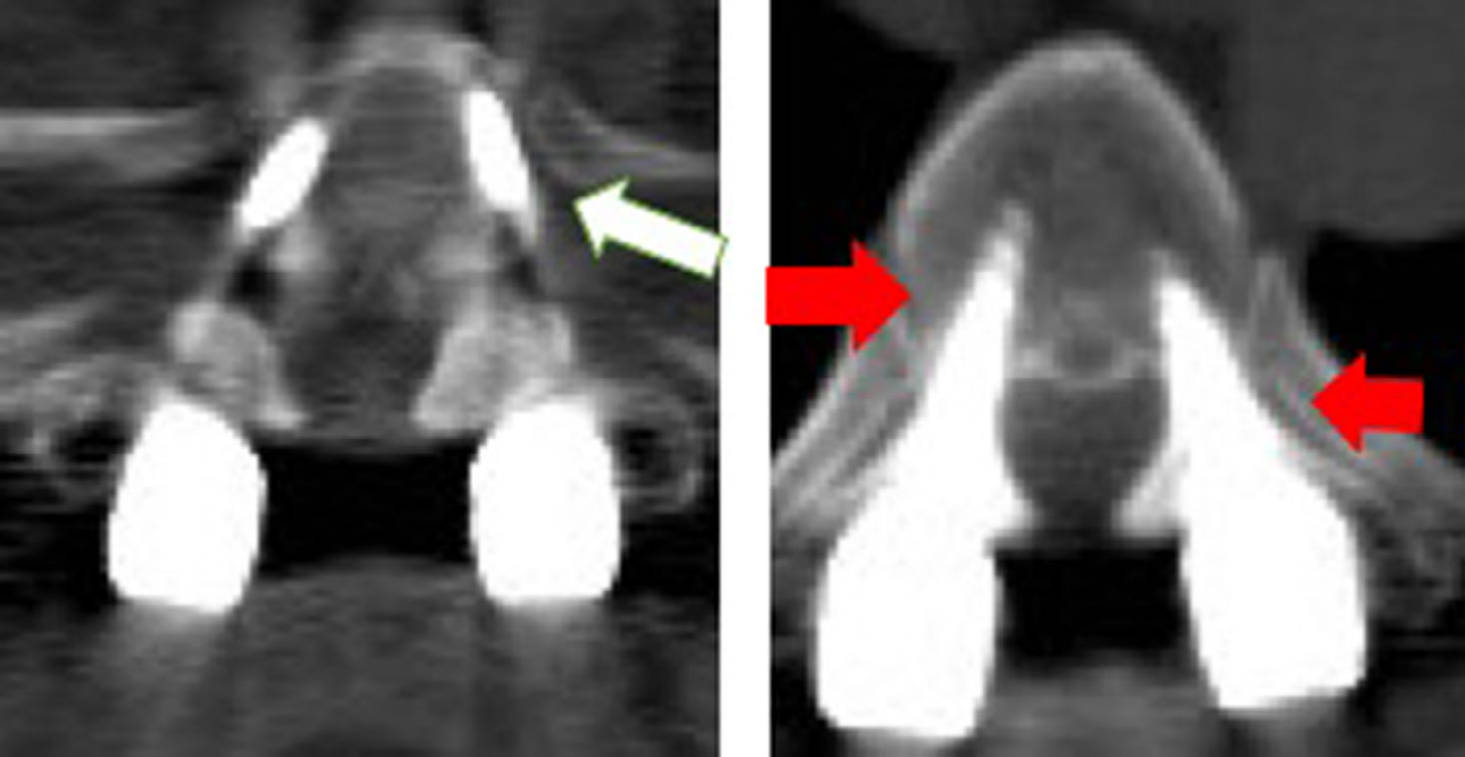
Inaccurate insert positioning: The white arrow indicates that the screw penetrates the cortex, and the red arrow indicates that the screw has penetrated the inner wall of the pedicle.

The Rampersaud criterion was used to evaluate the accuracy of pedicle screw. The Rampersaud criterion categorizes patients into four grades from A to D: A, no breach; B, less than 2 mm breach; C, 2–4 mm breach; D, more than 4 mm breach^18^. Grade A or B was considered accurate insert positioning based the concept of “safe‐zone”^19^, ^20^, ^21^ (Fig. 5), and grade C or D was considered as inaccurate insert positioning (Fig. 6).

#### *Immediate Postoperative Complications*

Immediate postoperative complications refer to a series of adverse events related to surgery that occur within 30 days after surgery. The index of immediate postoperative complications mainly includes: pulmonary embolism (PE) or other thromboembolic events, surgical site infection (SSI), neurovascular injury, and mortality.

### *Statistical Analysis*

Statistical analysis was performed using the Statistical Package for the Social Sciences (SPSS) version 18.0 (IBM Armonls, NY, USA). Numerical data are expressed by mean values ± standard deviation (SD). A *P* value of <0.05 was considered statistically significant. Student *t* test or chi‐squared test was performed to compared the mean values or data distribution.

## Results

### *Patients Demographics*

Patient demographic and comparison of the two surgery methods are shown in Tables 1 and 2. There were 24 males and 16 females, with an average age of 52.2 years (range, 24–77). There were 22 in the traditional group and 18 in the snake‐eye method group. And the age (mean ± SD) of traditional method group and snake‐eye method group was 53.77 ± 12.87 and 50.33 ± 10.48, respectively. The gender of traditional method group and snake‐eye method group was 15 males (68.18%) and nine males (50%), respectively. The traditional group had 12 (54.54%) obese people (BMI > 28 kg/m^2^), while the snake‐eye method group had 10 (55.56%). The traditional group had 14 (63.64%) people with high blood pressure, while the snake‐eye method group had 11 (61.11%). There were 16 (72.73%) smokers in the traditional group and 12 (66.67%) in the snake‐eye method group. The data baselines of the two groups were comparable because no impact of the two groups on population characteristics was demonstrated in the presented experiment.

**TABLE 1:** Patient demographic showed no significant differences between the two groups

Variables	Traditional method group	Snake‐eye method group	*P*‐value
Cases	22	18	—
Age (mean±SD, years)	53.77 ± 12.87	50.33 ± 10.48	0.3673
Sex [male (%)]	15 (68.18)	9 (50)	0.243
Obesity (BMI > 28 kg/m^2^) (%)	12 (54.54)	10 (55.56)	0.949
Smoking [Yes (%)]	16 (72.73)	12 (66.67)	0.677
Hypertension [Yes (%)]	14 (63.64)	11 (61.11)	0.641

**TABLE 2:** Time, accuracy and immediate (within 30 days after surgery) postoperative complication between the two groups

Variables	Traditional method group	Snake‐eye method group	*P*‐value
Time (mean ± SD, min)	5.34 ± 1.04	3.27 ± 0.896	<0.001
Accuracy (Rampersaud criterion)			
Grade A and B (%)	68 (62.96)	79 (85.87)	<0.001
Grade C and D (%)	40 (37.04)	13 (14.13)	
Compication			
PE	0	0	
SSI	2	1	0.714
NI	1	0	0.832
Mortality	0	0	

NI, neurovascular injury; PE, pulmonary embolism; SSI, surgical site infection.

### *Rampersaud Criterion*

**Fig. 7:**
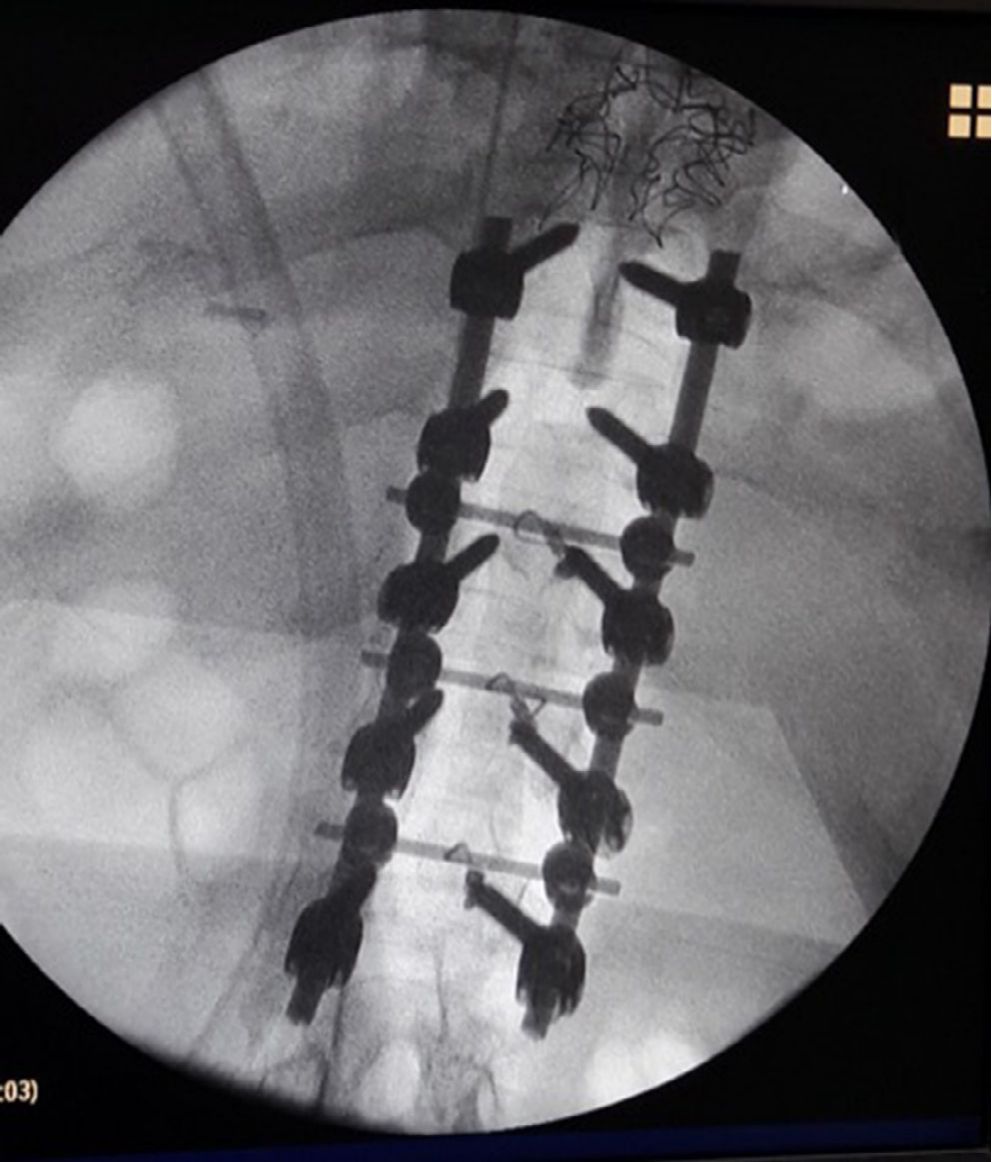
During the operation, C‐arm machine showed that the position of pedicle screw was accurate.

During the operation, C‐arm machine showed that the position of pedicle screw was accurate (Fig. 7). The time (mean ± SD) of traditional method group and snake‐eye method group was 5.34 ± 1.04 min and 3.27 ± 0.896 min, respectively, and the time of the two groups were different with statistical significance at the level of *P* = 0.05. According to Rampersaud criteria, the traditional group had 68 (62.96%) accurate insertions (grade A and B) of pedicle screws and 40 (37.04%) inaccurate insertions (grade C and D), while the snake‐eye method group had 79 (85.87%) accurate insertions (grade A and B) of pedicle screws and 13 (14.13%) inaccurate insertions (Grade C and D). There was a statistical difference in accuracy between the two groups at the level of *P* = 0.05.

### *Immediate Postoperative Complications*

**Fig. 8:**
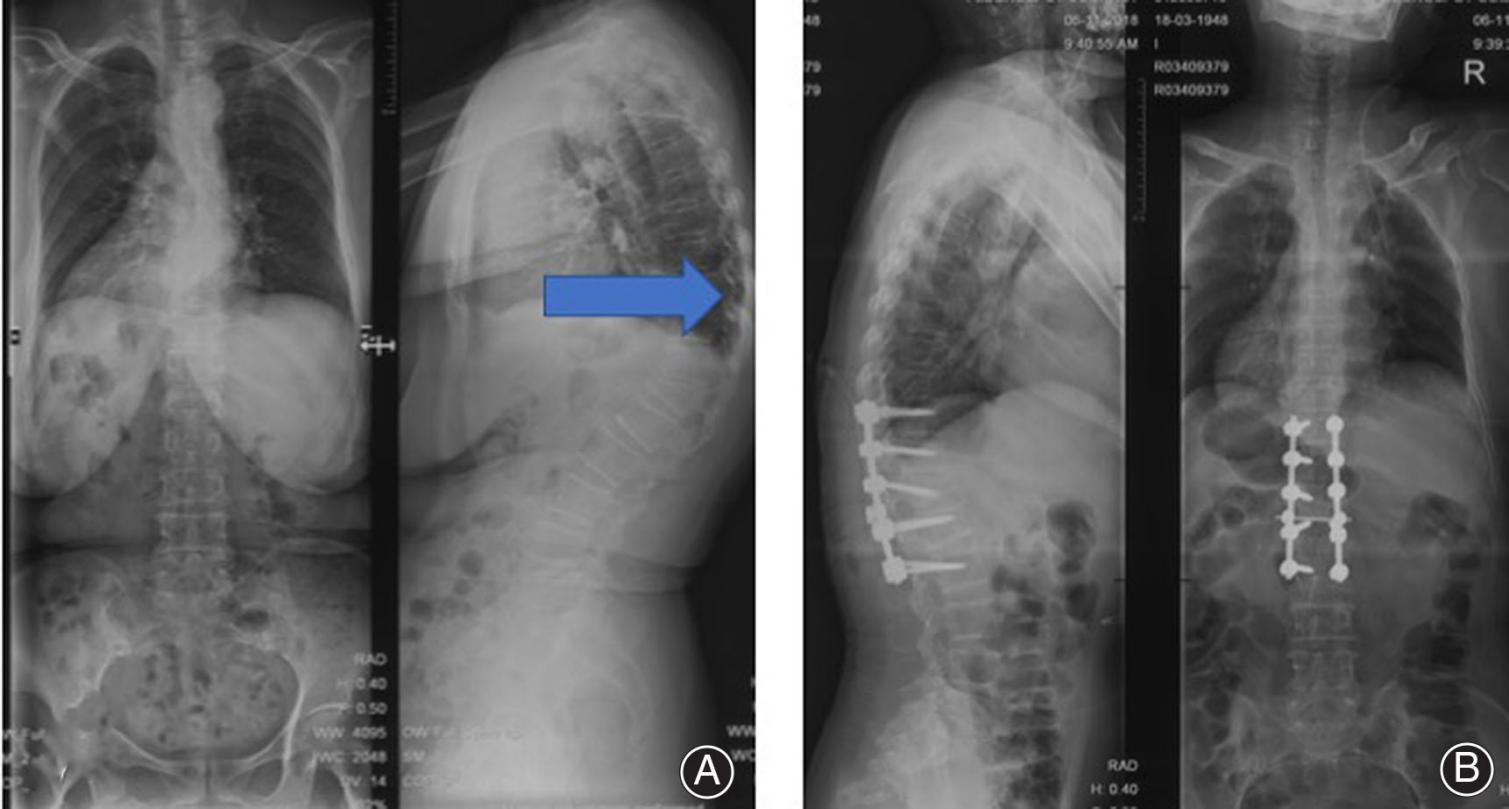
A case of kyphosis in a 52‐year‐old female treated with snake‐eye screwing. (A) Preoperative scoliosis was severe and compressed the viscera. (B) Postoperative correction scoliosis was satisfactory, screws were inserted along the axis of the pedicle, demonstrating that the pedicle screw placement was accurate.

The freehand technique has been proved to be safe and effective. We have applied the technique successfully in 40 patients, with minimal complications (Fig. 8). Pulmonary embolism (PE) and mortality were not detected, but we did find two cases of surgical site infection (SSI) and one case of neurovascular injury (NI) in the traditional method group, and one case of NI in the snake‐eye method group. All cases of SSI were performed with 0.5% iodophor and 75% alcohol and all patients improved gradually. We noticed that postoperative complications, including PE, SSI, NI, and mortality, in the two groups did not differ at the level of *P* = 0.05.

### *Typical Cases*

#### *Case 1*

A case of kyphosis in a 52‐year‐old female treated with snake‐eye screwing. Preoperative scoliosis was severe and compressed the viscera (Fig. 8A). The patient was treated with surgery for the correction of kyphosis. Postoperative correction scoliosis was satisfactory, screws were inserted along the axis of the pedicle, demonstrating that the pedicle screw placement was accurate and satisfied correction of kyphosis (Fig. 8B).

### *Case 2*

**Fig. 9:**
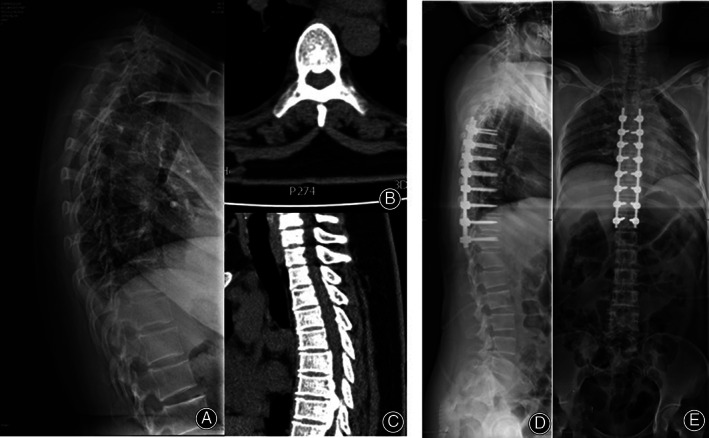
OPLL in a 47‐year‐old female with some neurological symptoms. (A, B, C) Preoperative radiographic images. The blue arrows indicate ossification of the posterior longitudinal ligament in the T_7‐9_ segments. (D, E) Postoperative X‐ray images.

A 47‐year‐old female with some neurological symptoms was finally diagnosed with ossification of posterior longitudinal ligaments (OPLL), and was treated with surgery. Preoperative radiographic images showed ossification of the posterior longitudinal ligament in the T_7‐9_ segments (Fig. 9A–C). Postoperative X‐ray images showed accurate insert of pedicle screws and spinal cord compression was relieved completely (Fig. 9D,E).

#### *Case 3*

**Fig. 10:**
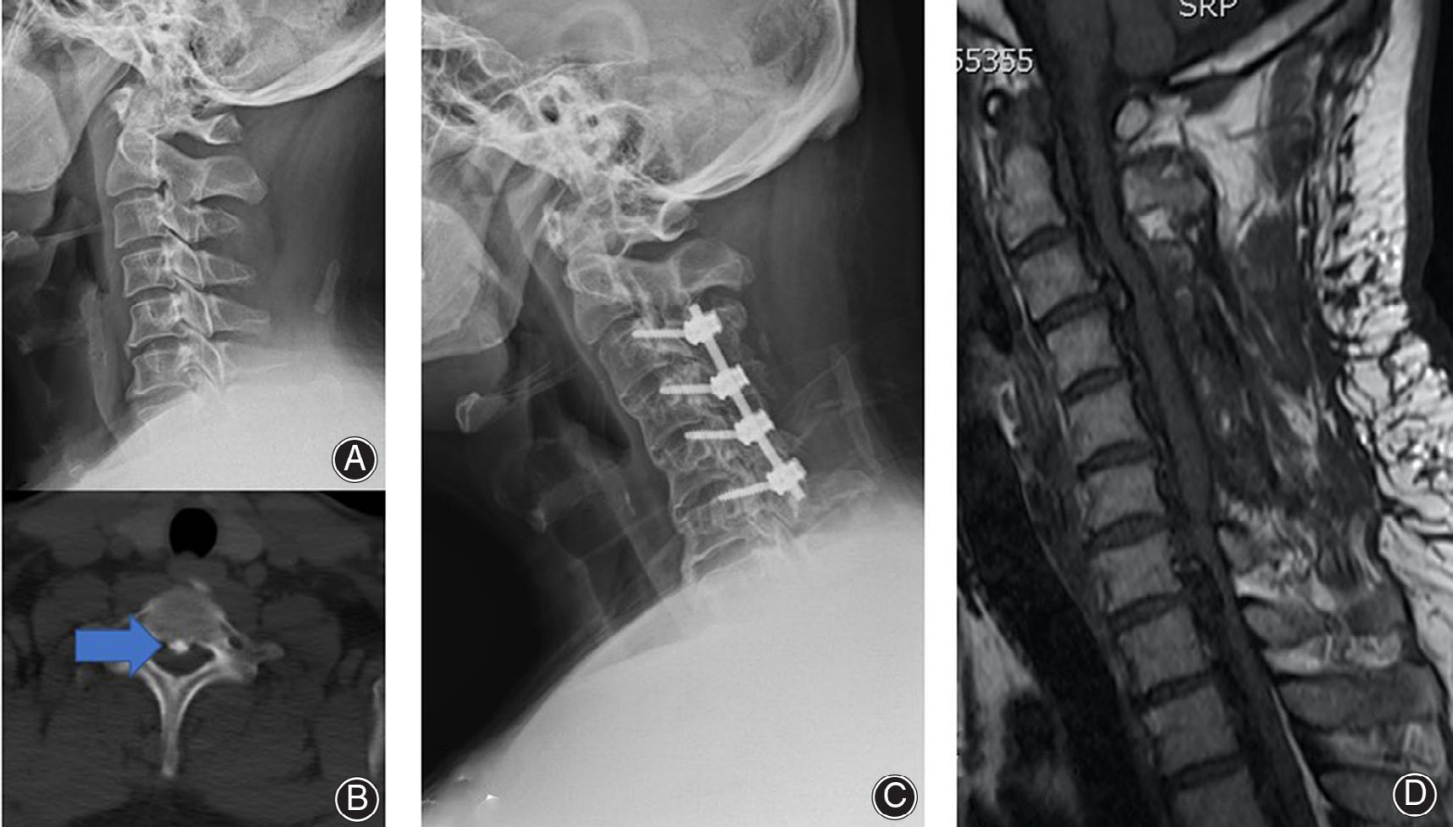
OPLL in a 34‐year‐old male with neurological symptoms. (A, B) Preoperative radiographic images. The blue arrows indicate ossification of the posterior longitudinal ligament in the C7 segment. (C) Postoperative X‐ray images showed pedicle screw position was accurate. (D) Postoperative MRI showed that spinal cord decompression was complete.

A 34‐year‐old male patient with neurological symptoms of OPLL. Preoperative radiographic images showed ossification of the posterior longitudinal ligament in the C_7_ segment (Fig. 10A,B). Postoperative X‐ray images showed pedicle screw position was accurate (Fig. 10C). Postoperative magnetic resonance imaging (MRI) showed that spinal cord decompression was complete (Fig. 10D).

## Discussion

### *Free‐hand Screwing and Computer‐assisted Screwing*

Free‐hand screwing is still the most commonly used way to achieve tri‐column stability from a posterior approach. Cervicothoracic screwing has always been a challenge, even for experienced spine surgeons. Our study found that the snake‐eye method was an effective free‐hand method to screw safely and quickly in the cervicothoracic region. Despite the small size of the cervicothoracic pedicles, the newly developed snake‐eye method could be effective in finding the correct pedicle trajactory without perforation. Advances have been made in intraoperative navigation systems, with newly applied ultrasound, or CT‐based navigation system, which increases the screwing accuracy and decreases complications, especially in patients with spine deformities. Yet, the system costs millions of dollars and few medical centers could afford the investment, which may inhibit the applications in hospitals from small‐ and medium‐scaled cities. Also, it might increase the screwing time when adjusting the machine and system. Free‐hand technique is still the most commonly used method to place screws, especially in emergency and limited situations. Studies have shown that there was no statistical significance between robot‐assist group and free‐hand group in accuracy and complication rate of pedicle screw placement^1^, ^18^. Since the navigation has difficulty in synchronizing with the cervical activities of the patient, Liu^22^ poses that manual screw placement is the more effective method.

### *Comparison of Entry Point*

The free‐hand procedure usually requires a clear understanding of the anatomy of cervicothoracic spine and a precise preoperative plan. Appropriate choice of entry point is the key to successful cervical pedicle screw placement. Various entry points have been demonstrated in previous reports. Abumi *et al*.^23^ selected the entrance point slightly lateral to the center of the lateral mass and close to the inferior edge of the inferior facet joint of the cranially adjacent vertebra, while Jeanneret *et al*.^10^ suggested that the entry point should be 3 mm beneath the facet joint on a vertical line in the middle of the articular mass. Lee *et al*.^24^ chose the entry point 2 mm medial to the lateral notch. According to Liu's study^22^, it is difficult to find the entry point during surgery when the vision is limited and anatomical structure is unclear, which may result in a higher risk of spinal cord injury caused by the screw entering the spinal canal. In our study, we set the entry point in the middle of the triangle formed by the lower edge of the superior articular facet, the medial edge of the transverse process, and the pars interarticularis. The entry point of snake‐eye method we develop in this study facilitates the removal of cortical bone and the exposure of cancellous bone with less variation in parameters of frame of reference.

### *Advantages of the Snake‐Eye Method*

One of the advantages of the snake‐eye method is that time of pedicle screw placement is short. In this study, the time of pedicle screw placement between snake‐eye method group and other groups are different with statistical significance at a level of *P* =0.05. The snake‐eye method is quick and convenient, which has more advantages in emergency conditions or without intraoperative navigation systems. Practically the snake‐eye method has lower learning curve which can be obtained by continuous practicing.

### *Anatomic Considerations*

A special blunt tap designed and manufactured by us was used to penetrate the vertebral pedicle gradually along the direction of the cancellous bone, reducing risks of screw perforation into the canal or outside the vertebra. This technique could significantly prevent iatrogenic cord injury and blood vessel lesions. Noticeably, the blunt drill (probe) could find the pedicle trajectory by itself and adjust the angulation when the bund head tough the stiff cortical bone. Parker *et al*.^25^ found that the accuracy rate of pedicle screw placement in thoracic spine (97.5%) was lower than that in lumbar spine (99.1%) with statistical significance at the level of *P* < 0.001, and T_4_ and T_6_ were estimated to be the levels with the highest incidence of screw perforation. Yukawa *et al*.^26^ reported that the proportion of lateral deviation of malpositioned screws reached 72%. We proposed some reasons for lateral perforation: (i) According to the research of Panjabi *et al*.^27^, the medial cortical shell (average value range: 1.2–2.0 mm) was measured to be 1.4 to 3.6 times as thick as the lateral cortical shell (average value range: 0.4–1.1 mm), so the lateral cortex is vulnerable to perforation. (ii) Screws are often inserted using a designed angle, however, screws tend to be inserted vertically relative to the pedicle because of the contractionary force of the paravertebral muscles. Complication assessment mainly includes: pulmonary embolism or other thromboembolic events, surgical site infection (SSI), neurovascular injury (NI), and mortality. Snake‐eye method did not significantly influence complication rates after spine surgery.

### *Limitation of the Study*

From the above discussion, we have attempted to introduce some concepts associated with a snake‐eye method based on fuzzy sets. However, this method we proposed in this study has its own limitations. Firstly, certain surgical experience is required to determine the location, which will undoubtedly increase the difficulty of surgery. Secondly, we had a small number of patients and insufficient follow‐up time, leading to a lack of judgment about the long‐term effects of snake‐eye method. However, we can be sure that this method has great advantages in fast nailing with bare hands, especially for nailing in emergency. Thus, first extension of the approach could be increased with experimental data and prolonged follow‐up time. In conclusion to this, it becomes obvious that the snake‐eye method is a novel and effective free‐hand technique of pedicle screw placement in cervicothoracic spine.

## Conclusion

This study highlights a safe and effective technique for pedicle screw placement in cervicothoracic spine. This technique may be particularly useful in emergency conditions with limited resources.

## Compliance with Ethical Standards

This research was approved by the ethics committee of Second Military Medical University.

## References

[1] Tian NF , Huang QS , Zhou P , *et al* Pedicle screw insertion accuracy with different assisted methods: a systematic review and meta‐analysis of comparative studies. Eur Spine J, 2011, 20: 846–859. 20862593 10.1007/s00586-010-1577-5 PMC3099151

[2] Xu RM , Ma WH , Wang Q , Zhao LJ , Hu Y , Sun SH . A free‐hand technique for pedicle screw placement in the lower cervical spine. Orthop Surg, 2009, 1: 107–112. 22009826 10.1111/j.1757-7861.2009.00023.x PMC6583121

[3] Kim YJ , Lenke LG , Bridwell KH , Cho YS , Riew KD . Free hand pedicle screw placement in the thoracic spine: is it safe?. Spine (Phila Pa 1976), 2004, 29: 333–342. 14752359 10.1097/01.brs.0000109983.12113.9b

[4] An HS , Wise JJ , Xu R . Anatomy of the cervicothoracic junction: a study of cadaveric dissection, cryomicrotomy, and magnetic resonance imaging. J Spinal Disord, 1999, 12: 519–525. 10598995

[5] Zhuang Z , Chen Y , Han H , *et al* Thoracic pedicle morphometry in different body height population: a three‐dimensional study using reformatted computed tomography. Spine (Phila Pa 1976), 2011, 36: 1547–1554. 21270680 10.1097/BRS.0b013e318210f063

[6] Boyle JJ , Singer KP , Milne N . Morphological survey of the cervicothoracic junctional region. Spine (Phila Pa 1976), 1996, 21: 544–548. 8852307 10.1097/00007632-199603010-00003

[7] Kreshak JL , Kim DH , Lindsey DP , Kam AC , Panjabi MM , Yerby SA . Posterior stabilization at the cervicothoracic junction: a biomechanical study. Spine (Phila Pa 1976), 2002, 27: 2763–2770. 12486344 10.1097/00007632-200212150-00005

[8] Ames CP , Bozkus MH , Chamberlain RH , *et al* Biomechanics of stabilization after cervicothoracic compression‐flexion injury. Spine (Phila Pa 1976), 2005, 30: 1505–1512. 15990664 10.1097/01.brs.0000167824.19875.e9

[9] Abumi K , Kaneda K . Pedicle screw fixation for nontraumatic lesions of the cervical spine. Spine (Phila Pa 1976), 1997, 22: 1853–1863. 9280021 10.1097/00007632-199708150-00010

[10] Jeanneret B , Gebhard JS , Magerl F . Trans‐pedicular screw fixation of articular mass fracture‐separation. J Spinal Disord, 1994, 7: 222–229. 7919645 10.1097/00002517-199407030-00004

[11] Ebraheim N . Anatomic relations of the thoracic pedicle to the adjacent neural structures. Spine (Phila Pa 1976), 1997, 22: 1553–1556. 9253087 10.1097/00007632-199707150-00002

[12] Karaikovic EE , Yingsakmongkol W , Gaines RW . Accuracy of cervical pedicle screw placement using the funnel technique. Spine (Phila Pa 1976), 2001, 26: 2456–2462. 11707710 10.1097/00007632-200111150-00012

[13] Gertzbein SD , Robbins SE , Belmont PJ . Accuracy of pedicular screw placement in vivo. Spine (Phila Pa 1976), 1990, 15: 11–14. 2326693 10.1097/00007632-199001000-00004

[14] Gelalis ID , Paschos NK , Pakos EE , *et al* Accuracy of pedicle screw placement: a systematic review of prospective in vivo studies comparing free hand, fluoroscopy guidance and navigation techniques. Eur Spine J, 2012, 21: 247–255. 21901328 10.1007/s00586-011-2011-3 PMC3265579

[15] Castro HM , Halm H , Jerosch J , *et al* Accuracy of pedicle screw placement in lumbar vertebrae. Spine (Phila Pa 1976), 1996, 21: 1320–1324. 8725923 10.1097/00007632-199606010-00008

[16] Schwarzenbach O , Berlemann U , Jost B , *et al* Accuracy of computer‐assisted pedicle screw placement: an in vivo computed tomography analysis. Spine (Phila Pa 1976), 1997, 22: 452–458. 9055375 10.1097/00007632-199702150-00020

[17] Errani C , Vanel D , Gambarotti M , Alberghini M , Picci P , Faldini C . Vascular bone tumors: a proposal of a classification based on clinicopathological, radiographic and genetic features. Skeletal Radiol, 2012, 41: 1495–1507. 22993209 10.1007/s00256-012-1510-6

[18] Rampersaud YR , Pik JT , Salonen D , *et al* Clinical accuracy of fluoroscopic computer‐assisted pedicle screw fixation: a CT analysis. Spine (Phila Pa 1976), 2005, 30: 183–190. 10.1097/01.brs.0000157490.65706.38 15803068

[19] Schizas C , Thein E , Kwiatkowski B , Kulik G . Pedicle screw insertion: robotic assistance versus conventional C‐arm fluoroscopy. Acta Orthop Belg, 2012, 78: 240–245. 22696996

[20] Lonjon N , Chan‐Seng E , Costalat V , Bonnafoux B , Vassal M , Boetto J . Robot‐assisted spine surgery: feasibility study through a prospective case‐matched analysis. Eur Spine J, 2016, 25: 947–955. 25575857 10.1007/s00586-015-3758-8

[21] Laudato PA , Pierzchala K , Schizas C . Pedicle screw insertion accuracy using O‐Arm, robotic guidance, or freehand technique: a comparative study. Spine (Phila Pa 1976), 2018, 43: 373–378. 10.1097/BRS.0000000000002449 29019807

[22] Liu J , Li Y , Wu Y , *et al* A novel method of cervical pedicle screw placement from C3 to C5 and its clinical applications. Spine (Phila Pa 1976), 2013, 38: 504–512. 10.1097/BRS.0b013e3182880065 23354110

[23] Abumi K . Transpedicular screw fixation for traumatic lesions of the middle and lower cervical spine: description of the techniques and preliminary report. J Spinal Disord, 1994, 7: 19–28. 8186585 10.1097/00002517-199407010-00003

[24] Lee DH , Lee SW , Kang SJ , *et al* Optimal entry points and trajectories for cervical pedicle screw placement into subaxial cervical vertebrae. Eur Spine J, 2011, 20: 905–911. 21475996 10.1007/s00586-010-1655-8 PMC3099155

[25] Parker SL , Mcgirt MJ , Farber SH , *et al* Accuracy of free‐hand pedicle screws in the thoracic and lumbar spine: analysis of 6816 consecutive screws. Neurosurgery, 2011, 68: 170–178. 21150762 10.1227/NEU.0b013e3181fdfaf4

[26] Yukawa Y , Kato F , Ito K , *et al* Placement and complications of cervical pedicle screws in 144 cervical trauma patients using pedicle axis view techniques by fluoroscope. Eur Spine J, 2009, 18: 1293–1299. 19488794 10.1007/s00586-009-1032-7 PMC2899535

[27] Panjabi MM , Shin EK , Chen NC , Wang JL . Internal morphology of human cervical pedicles. Spine (Phila Pa 1976), 2000, 25: 1197–1205. 10806495 10.1097/00007632-200005150-00002

